# Functions of FUS/TLS From DNA Repair to Stress Response: Implications for ALS

**DOI:** 10.1177/1759091414544472

**Published:** 2014-08-26

**Authors:** Reddy Ranjith Kumar Sama, Catherine L. Ward, Daryl A. Bosco

**Affiliations:** 1 Department of Neurology, University of Massachusetts Medical School, Worcester, MA, USA; 2 Department of Biochemistry and Molecular Pharmacology, University of Massachusetts Medical School, Worcester, MA, USA

**Keywords:** FUS/TLS, amyotrophic lateral sclerosis, DNA damage repair, stress response, RNA processing, stress granules

## Abstract

Fused in sarcoma/translocated in liposarcoma (FUS/TLS or FUS) is a multifunctional DNA-/RNA-binding protein that is involved in a variety of cellular functions including transcription, protein translation, RNA splicing, and transport. FUS was initially identified as a fusion oncoprotein, and thus, the early literature focused on the role of FUS in cancer. With the recent discoveries revealing the role of FUS in neurodegenerative diseases, namely amyotrophic lateral sclerosis and frontotemporal lobar degeneration, there has been a renewed interest in elucidating the normal functions of FUS. It is not clear which, if any, endogenous functions of FUS are involved in disease pathogenesis. Here, we review what is currently known regarding the normal functions of FUS with an emphasis on DNA damage repair, RNA processing, and cellular stress response. Further, we discuss how ALS-causing mutations can potentially alter the role of FUS in these pathways, thereby contributing to disease pathogenesis.

## Introduction

**Figure 1.**
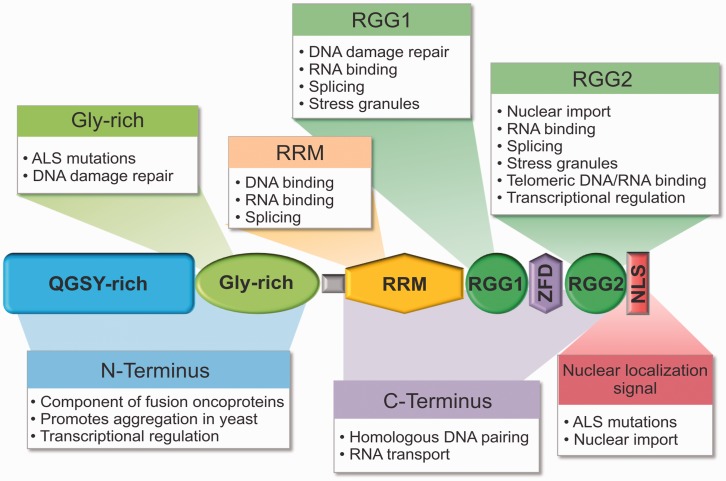
The functional domains within fused in sarcoma (FUS). FUS binds DNA, RNA, and proteins to perform a diverse array of functions. Summarized here are the known functions of FUS annotated onto the domain structure of the protein. *Note.* QGSY-rich = glutamine-glycine-serine-tyrosine-rich or prion-like domain; Gly-rich = glycine-rich; RGG = arginine-glycine-glycine-rich; RRM = RNA recognition motif; ZFD = zinc finger domain; NLS = nuclear localization signal; ALS = amyotrophic lateral sclerosis.

Fused in sarcoma/translocated in liposarcoma (FUS/TLS or FUS) belongs to the FET (previously TET) family of proteins, which also includes EWS (Ewing sarcoma), TAF15 (TATA box-binding protein-associated factor 68 kDa), and the *Drosophila* homolog SARFH (sarcoma-associated RNA-binding fly homolog; Law et al., 2006; Tan & Manley, 2009a). This family represents a rare class of proteins that function at all stages of gene expression from transcription to protein translation. Moreover, FET proteins carry out numerous roles by interacting with DNA, RNA, and proteins. The diverse functional interactions of FET proteins are driven by their conserved, albeit complex, structures containing an N-terminal glutamine-glycine-serine-tyrosine (QGSY)-rich (prion-like) domain, glycine-rich region, RNA-recognition motif (RRM), zinc-binding domain, and C-terminal arginine-glycine-glycine (RGG)-rich domains (Figure 1). FET proteins are ubiquitously expressed in most tissues and are predominantly localized to the nucleus of cells (Andersson et al., 2008), although they engage in nucleocytoplasmic shuttling (Zinszner et al., 1997) and thus play important roles in both compartments. Although these proteins share overlapping functions in DNA- and RNA-associated processes, they also have unique roles in the cell (Blechingberg et al., 2012; Kovar, 2011; Law et al., 2006; Riggi et al., 2007). Herein, we will focus on the normal functions of FUS and the aberrant role of this protein in neurodegeneration.

FUS was first identified in the context of a chimeric oncoprotein in myxoid liposarcomas (MLS). In MLS and other cancers, chromosomal translocation events result in aberrant transcription factors, formed by a fusion between the N-terminus of FUS and the DNA-binding domain of an endogenous transcription factor such as CHOP (C/EBP homology protein; Crozat, Aman, Mandahl, & Ron, 1993; Rabbitts et al., 1993), ERG (ETS-related gene; Ichikawa et al., 1994; Panagopoulos et al., 1994; Shing et al., 2003), ATF1 (activation transcription factor 1; Raddaoui et al., 2002; Waters et al., 2000), and BBF2H7 (BBF2 human homolog on chromosome 7; Storlazzi et al., 2003). These and other FET oncoproteins account for nearly half of the fusion proteins involved in the pathogenesis of sarcomas (Riggi et al., 2007).

FUS has recently been linked to amyotrophic lateral sclerosis (ALS, also known as Lou Gehrig’s disease; Kwiatkowski et al., 2009; Vance et al., 2009) and frontotemporal lobar degeneration (FTLD; Munoz et al., 2009; Neumann, Rademakers, et al., 2009; Neumann et al., 2009; Urwin et al., 2010), two related yet distinct neurodegenerative disorders (Rademakers et al., 2012). ALS is a progressive motor neuron disease that culminates in paralysis and death within 3 to 5 years of symptom onset. A majority (∼90%) of ALS cases are sporadic in nature with an unknown etiology, while the remaining ∼10% of cases are attributed to inheritable genetic defects (Sreedharan & Brown, 2013). Mutations in the gene encoding FUS account for ∼3% to 5% of inherited, or familial, ALS (FALS). To date, it is not clear whether ALS-linked mutations cause a loss of normal FUS function or induce the protein to acquire a gain of toxic function in the context of this disease. FTLD is characterized by progressive decline in behavior, personality, or language, symptoms that are attributed to the degeneration of the frontal and temporal lobes. Twenty-five percent to 50% of cases have a family history, and disease pathology is often characterized by neuronal inclusions of disease-specific proteins (Rademakers et al., 2012). Although FUS pathology is detected in both FALS-FUS and FTLD-FUS, the majority of disease-causing mutations within FUS are associated with FALS-FUS cases. Therefore, we will focus our discussion on the mechanism of mutant FUS in the context of FALS.

## Reading and Repairing the Genetic Code

**Figure 2.**
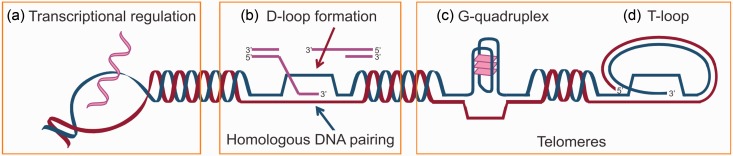
FUS directly binds DNA. (a) FUS binds the promoters of >1,000 genes, indicative of a role in transcriptional regulation. (b) FUS binds both single- and double-stranded DNA and is important for two critical steps in homologous recombination: D-loop formation and homologous DNA pairing. When a double-strand break occurs in DNA, the 5′ end of the break is trimmed back to create a 3′ overhang of single-stranded DNA. This 3′ single-stranded DNA then binds a complementary sequence within duplex DNA of a homologous chromosome or sister chromatid, a process called strand invasion (reviewed in X. Li & Heyer, 2008). (c) FUS binds G-quadruplexes in telomeres. (d) Analogous to the role of FUS in D-loop formation, FUS may also be important for T-loop formation at the ends of telomeres. T-loops are formed when a single-stranded, G-rich DNA overhang at the end of a chromosome forms a loop and anneals to a complementary 5′ C-rich sequence (Griffith et al., 1999; reviewed in Greider, 1999).

Interactions between FUS and DNA underlie several putative functions of FUS in the context of DNA processing (Figure 2). For example, FUS directly binds both single- and double-stranded DNA (Baechtold et al., 1999; Liu et al., 2013), localizes to RNAPII promoters (Tan et al., 2012) and telomeres (Dejardin & Kingston, 2009; Takahama et al., 2009), and is associated with higher order DNA structures (Baechtold et al., 1999; Takahama & Oyoshi, 2013; Takahama et al., 2013). FUS was implicated in transcriptional regulation (Figure 2(a)) and DNA damage response before the discovery of FUS in ALS; however, recent literature provides considerable insight into how FUS functions in these processes and, importantly, how ALS-linked mutations in FUS alter these functions.

### FUS Directly Binds and Repairs DNA

The discovery that FUS and hPOMp75 were in fact the same protein provided an early clue that FUS plays a direct role in DNA repair. Homologous DNA pairing is an essential step in the repair of double-stranded DNA breaks by homologous recombination (HR; Figure 2(b)). In the pairing on membrane (POM) assay, FUS/hPOMp75 promoted binding between duplex DNA and a homologous single-stranded DNA probe affixed to nitrocellulose membrane (Akhmedov et al., 1995; Bertrand et al., 1999). D-loop formation (Figure 2(b)) was also correlated with FUS concentration (Baechtold et al., 1999). Interestingly, the FUS-CHOP oncoprotein containing the N-terminus of FUS (residues 1–268) fused to the CHOP transcription factor lacks this homologous DNA-pairing capability (Baechtold et al., 1999), implicating the C-terminal region of FUS in homologous DNA pairing. This notion is supported by recent in vitro binding experiments demonstrating that a FUS fragment (residues 278–385) containing the RRM domain binds DNA with micromolar binding affinity (Liu et al., 2013). The DNA-binding zinc finger motif within the C-terminal region of FUS is another region that potentially binds DNA (Figure 1). That a majority of ALS-causing mutations are located within the C-terminus of FUS raises the intriguing possibility that these mutations interfere with the DNA-pairing function of FUS, potentially compromising the stability of the genome.

Homologous DNA pairing is also required for the formation of T-loops, telomeric structures that are similar to D-loops (Figure 2(d)). The formation of T-loops protects the ends of telomeres and allows the cell to distinguish between the end of chromosomes and sites of DNA damage (reviewed in de Lange, 2004). In light of the evidence that FUS facilitates D-loop formation and binds telomeric DNA (Dejardin & Kingston, 2009; Takahama et al., 2009, 2013), it is tempting to speculate that FUS also aids in T-loop formation. Although not mutually exclusive, FUS may influence telomere length through interactions outside of the T-loop. A recent report shows that the RGG domain of FUS binds G-quadruplex structures (Figure 2(c)) in both telomeric DNA and noncoding telomeric RNA (termed TERRA; Takahama et al., 2013). The authors posit that FUS modulates telomere length through a mechanism involving histone methylation (Takahama et al., 2013), which is important for recombination events that maintain telomere length (Benetti et al., 2007). In fact, they demonstrated that FUS binds SUV4-20H2, a histone methyltransferase, and that overexpression of FUS causes increased histone methylation and telomere shortening (Takahama et al., 2013). Dysfunctional telomeres have been pathologically linked to Alzheimer’s disease and Parkinson’s disease and may also be relevant to other neurodegenerative diseases (reviewed in Zhu et al., 2011).

**Figure 3.**
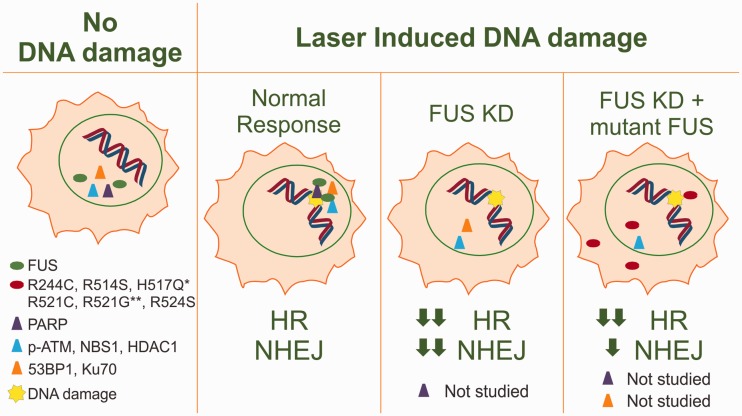
FUS is recruited to sites of DNA damage and contributes to DNA-damage repair. Under normal conditions, FUS (green oval) and common repair proteins (triangles) localize to sites of laser-induced DNA damage (yellow star). Under conditions of FUS knockdown, these repair proteins are not recruited to sites of DNA damage and the efficiency of both homologous recombination and nonhomologous end joining is reduced. Mutant FUS (red ovals) is still able to localize to sites of damage in the absence of endogenous FUS (**discrepancy in the literature for the degree of localization of variant R521G). Exogenous mutant FUS does not fully rescue DNA-damage repair when endogenous FUS is knocked-down (*exception, FUS H517Q), although mutant FUS is able to recover NHEJ to a greater extent than HR (*NHEJ is fully recovered by FUS H517Q). *Note.* FUS = fused in sarcoma; KD = knockdown; PARP = adenosine diphosphate [ADP] ribose polymerase; HR = homologous recombination; NHEJ = nonhomologous end joining; p-ATM = phosphorylated-ataxia telanogiectasia mutated; NBS1 = Nijmegen breakage syndrome-1; HDAC = histone deacetylase 1; 53BP1 = p53-binding protein 1.

Although early studies showed that FUS promotes homologous DNA pairing and the formation of D-loops, it remained unclear at the time whether FUS was required for DNA damage repair. Several studies have recently shed light on this important question (Figure 3). For example, it was shown that FUS localizes to sites of laser-induced DNA damage within human osteosarcoma U2OS (Mastrocola et al., 2013; W. Y. Wang et al., 2013) and human lung adenocarcinoma epithelial (A549) cells (Rulten et al., 2013). For this assay, cells were microirradiated with a 405-nm diode laser, and the nuclear localization of FUS was monitored by fluorescence microscopy. The redistribution of FUS to sites of DNA damage occurs prior to that of other key DNA-repair proteins, including NBS1 (Nijmegen breakage syndrome-1), p-ATM (phosphorylated-ataxia telanogiectasia mutated), γH2AX (phosphorylated histone 2 A.X), and Ku70 (W. Y. Wang et al., 2013). An upstream role for FUS in DNA damage response was further demonstrated by the reduced localization of these proteins to DNA lesions when FUS expression was knocked down (W. Y. Wang et al., 2013).

Using two established assays for double-stranded DNA repair, one that measures HR activity and the other nonhomologous end joining (NHEJ), FUS was found to be required for efficient double-strand break repair (Mastrocola et al., 2013; W. Y. Wang et al., 2013). In the absence of FUS, the efficiency of both HR and NHEJ was decreased 30% to 50%. This reduction was thought to be significant considering that ∼50% reduction in these repair processes was observed when the expression of known DNA-repair proteins was reduced (Mastrocola et al., 2013; W. Y. Wang et al., 2013). In primary mouse cortical neurons, cells that mainly utilize the NHEJ pathway for DNA double-strand break repair (Sharma, 2007; reviewed in Rao, 2007), FUS depletion resulted in an ∼65% to 80% reduction in NHEJ efficiency and increased levels of damaged DNA as determined by the Comet assay (W. Y. Wang et al., 2013). Despite this increase in damaged DNA, the authors detected a deficiency in the recruitment of two common protein markers of double-stranded DNA break sites, γH2AX and 53BP1 (p53-binding protein 1; W. Y. Wang et al., 2013). Together, these findings demonstrate that FUS plays an important role in the initiation and efficiency of DNA damage repair processes in both proliferating cells and in postmitotic, nonproliferating cells such as neurons.

Both PARP (poly adenosine diphosphate [ADP] ribose polymerase) and HDAC1 (histone deacetylase 1) are proteins implicated in the mechanism(s) associated with the localization of FUS at double-stranded DNA breaks. While PARP is a known regulator of DNA damage repair (reviewed in De Vos et al., 2012; Schreiber et al., 2006), HDAC1 was only recently shown to play a role in DNA damage response (Dobbin et al., 2013; Miller et al., 2010; Thurn et al., 2013). The role of HDAC1 in DNA repair is not yet elucidated, but it appears that HDAC1 and FUS function together to repair double-stranded DNA breaks. In support of this view, the levels of HDAC1 at sites of laser-induced DNA damage was reduced in cells when FUS expression was knocked down (W. Y. Wang et al., 2013; Figure 3). A similar association has been observed between FUS and PARP. During the cellular response to DNA damage, PARP binds DNA at sites of single-strand breaks, where it polymerizes a poly adenosine diphosphate ribose (PAR) chain that signals the recruitment of various DNA repair proteins (reviewed in De Vos et al., 2012; Schreiber et al., 2006). FUS associates with PAR chains in vitro (Mastrocola et al., 2013; Rulten et al., 2013) via its RGG2 domain, which is also sufficient to recruit FUS to DNA lesions in cultured cells (Mastrocola et al., 2013). Further, inhibition of PARP activity prevented the recruitment of FUS to laser-induced double-stranded DNA breaks (Mastrocola et al., 2013; Rulten et al., 2013). In contrast, inactivation of either ATM or DNA-PK, two other key regulators of the DNA damage response, had no effect on the recruitment of FUS to laser-induced DNA damage (Mastrocola et al., 2013). Thus, while ATM can phosphorylate FUS in response to double-stranded DNA breaks (Gardiner et al., 2008), this association does not appear to be required for the recruitment of FUS to these sites.

### DNA Damage Repair in ALS-FUS

A critical goal in the ALS field is to determine whether ALS-linked mutations in FUS impair DNA damage repair and whether such defects play a role in disease pathogenesis. Studies with several ALS-linked variants in the laser-induced DNA damage assay discussed earlier have produced conflicting results: R244C, R514S, H517Q, R521C (W. Y. Wang et al., 2013), R521G, and R524S (Mastrocola et al., 2013) were recruited to sites of laser-induced DNA damage in U2OS cells to a similar degree as FUS WT, whereas in A549 cells, recruitment of R521G was reduced relative to FUS WT (Rulten et al., 2013; Figure 3). Whether these discrepancies are due to cell type or due to variability in assay conditions between laboratories is unclear.

W. Y. Wang et al. (2013) knocked down the expression of endogenous FUS in U2OS cells and directly examined the ability of several ALS-linked variants to perform either HR- or NHEJ-mediated DNA repair. All of the FUS variants tested (R244C, R514S, H517Q, and R521C) were deficient in HR-mediated DNA repair relative to FUS WT (Figure 3). Interestingly, there is no correlation between HR activity and the nuclear/cytoplasmic localization of FUS variants. For example, FUS R244C is predominately expressed in the nucleus and yet exhibited the most pronounced defect in HR, indicating that the loss of function phenotype with respect to HR is not simply due to the absence of FUS from the nucleus. Overall, the impaired role of FUS variants in DNA repair was more pronounced in HR than NHEJ, where in the latter pathway FUS H517Q fully rescued the loss of WT FUS expression (W. Y. Wang et al., 2013).

The implications of these observations for ALS-FUS are that an accumulation of damaged DNA in motor neurons could eventually lead to cell death. Damaged DNA accumulates normally as a function of age, presumably due to a lifetime exposure to DNA-damaging agents and a decline in quality control pathways (reviewed in Gorbunova et al., 2007). Defects in DNA repair due to FUS mutations are therefore expected to manifest in adulthood, which coincides with the mean age of onset (∼55 years) for ALS-FUS. Neurons may be particularly susceptible to accumulated DNA damage, as they lack the ability to replicate and self-renew. In support of this hypothesis, postmortem brain sections from the motor cortex of ALS patients harboring either FUS R521C or P525L mutations display increased levels of the γH2AX DNA damage marker relative to control brain sections (W. Y. Wang et al., 2013). However, it should be noted that γH2AX levels also correlate with apoptosis (Rogakou et al., 2000), which is an established cell death pathway in both ALS and related disorders (Pasinelli & Brown, 2006). Therefore, an alternative interpretation of these data is that there are increased levels of apoptosis in these end-stage diseased tissues, which is to be expected. Transgenic rodent models expressing ALS-linked FUS mutants may be better suited to investigate defects in the DNA damage response and repair pathways as a function of both mutant FUS expression and age. In fact, a new transgenic mouse expressing FUS R521C that exhibits severe motor defects and death 4 to 6 weeks after symptom onset also exhibits elevated levels of DNA damage markers (e.g., γH2AX, phosphorylated p53, and ATF3) in the CNS (Qiu et al., 2014). Comet assays performed on isolated neurons supported this observation, with >50% of neurons from R521C mice demonstrating comet tails compared with ∼20% from nontransgenic control littermates. However, these neurons lacked cleaved caspase-3 signal and were TUNEL-negative, indicating that damaged DNA and motor neuron death do not result from apoptosis (Qiu et al., 2014). Together, this evidence supports a disruption of DNA damage repair as a function of mutant FUS expression.

Supporting the notion that FUS plays a key role in maintaining DNA integrity, two FUS knockout (*FUS−/−*) mouse models reveal signs of genomic instability. Kuroda et al. (2000) generated a *FUS−/−* mouse model that displays a normal life span, but is smaller in size, and exhibits male sterility and reduced female fertility. These mice, and the embryonic fibroblasts derived from them, are more sensitive to ionizing irradiation (Kuroda et al., 2000). Similarly, embryonic fibroblast cells derived from the *FUS−/−* model reported by Hicks et al. (2000) exhibited a high incidence (67%) of aneuploidy and frequent observations of chromosomal breakage. That *FUS+/−* mice behaved similar to *FUS+/+* mice (Kuroda et al., 2000) indicates the sufficiency of one functional copy of FUS to facilitate a normal response to ionizing irradiation. This observation disputes a loss of FUS function mechanism for autosomal-dominant ALS-linked mutations in FUS such as those employed in the DNA-repair assays described earlier (W. Y. Wang et al., 2013), unless these variants exhibit a dominant negative effect, as suggested by the FUS-R521C transgenic mouse model where mutant FUS formed a stable complex with endogenous FUS, affecting its localization and interfering with binding partners (Qiu et al., 2014). It is important to remember, however, that both *FUS−/−* mouse models were reported 9 years before the first reports of FUS in ALS (Kwiatkowski et al., 2009; Vance et al., 2009); while paralysis of *FUS−/−* mice would be obvious, it is unlikely that these *FUS−/−* mice were monitored for more subtle signs of motor neuron degeneration. In addition to ALS animal models, motor neurons derived from human-derived induced pluripotent stem cells (iPSCs) could be used to explore the role of DNA damage in ALS pathogenesis, particularly whether motor neurons harboring ALS-linked FUS mutations are more susceptible to DNA-damaging agents.

### The Role of FUS in Transcriptional Regulation

In addition to playing a direct role in DNA repair at the sites of damage, FUS may also promote genomic integrity through its role as a transcriptional regulator. For example, FUS interacts with PGC-1α (proliferator-activated receptor γ-coactivator 1α), as shown in a yeast two-hybrid screen and confirmed in mammalian 293 T cells (Sanchez-Ramos et al., 2011). PGC-1α is a transcriptional coactivator of oxidative stress protection genes. A reduction of PGC-1α activity with a concomitant increase in reactive oxidative species (ROS) was observed in *FUS−/−* MEFs (Sanchez-Ramos et al., 2011). Keeping ROS levels in check may be particularly important for motor neurons due to their high metabolic activity. A prime target of ROS is DNA. The most common DNA lesion in neurons results from oxidation of nucleic acids, such as 8-hydroxy-2′-deoxy-guanosine (8-oxo-G) species, which can be repaired through the base-excision repair (BER) pathway (reviewed in Coppede, 2011). Postmortem patient tissues of Alzheimer’s disease, Parkinson’s disease (Jeppesen, Bohr, & Stevnsner, 2011), and ALS (Coppede, 2011) show elevated levels of DNA damage, including 8-oxo-G, and decreased activity of DNA repair enzymes. That oxidative stress is a pathological hallmark of these and other neurodegenerative disorders suggests that DNA damage arising from ROS may represent a general downstream consequence of disease pathogenesis, not necessarily due to defects in FUS function. Nonetheless, in the context of ALS-FUS and FTLD-FUS, defective DNA repair as a consequence of FUS dysfunction (W. Y. Wang et al., 2013) may be compounded by increased levels of ROS resulting from reduced expression of ROS protection genes.

FUS may also influence DNA damage response through transcriptional regulation of cell cycle arrest genes. Cell cycle arrest occurs in response to DNA damage to allow for DNA repair, thus preventing the propagation of damaged DNA (reviewed in B. B. Zhou & Elledge, 2000). In response to DNA damage induced by ionizing radiation, FUS is recruited to the cyclin D1 (*CCND1*) promoter and inhibits the expression of cyclin D1 (X. Wang et al., 2008), a key regulator of cell cycle progression (Fu et al., 2004). Through an interaction modulated by noncoding RNA, FUS was shown to bind and inhibit the histone acetyltransferase activity of the transcriptional coactivators CREB-binding protein and p300, thus reducing transcription of *CCND1* and halting the cell cycle (X. Wang et al., 2008). Although neurons are postmitotic and do not normally progress through the cell cycle, accumulating evidence suggests that inappropriate cell cycle reentry induces apoptosis and may therefore be a relevant mechanism in neurodegeneration (Bonda et al., 2010; Herrup & Yang, 2007). In Alzheimer’s disease, for instance, neurons exhibit increased levels of cell cycle markers long before the classic signs of this disease, such as amyloid-β plaques (reviewed in Bonda et al., 2010; Herrup, 2012). The cyclin D/CDK4,6 complex is responsible for reinitiating the cell cycle (Sherr, 1994); interestingly, increased expression of cyclin D and cdk4 is evident in motor neurons of ALS mice and patient tissue (Nguyen et al., 2003; Ranganathan & Bowser, 2003). One could speculate that dysregulation of *CCND1* expression in ALS-FUS could inappropriately reinitiate the cell cycle and trigger apoptosis. To date, the regulation of *CCND1* by FUS has only been demonstrated in macrophage cells (X. Wang et al., 2008) and prostate cancer cells (Brooke et al., 2010) and remains to be investigated in neurons.

The role of FUS in transcription extends well beyond PGC-1α and cyclin D1 (reviewed in Law et al., 2006; Tan & Manley, 2009a). FUS also interacts with other transcription factors and regulates the expression of their target genes (Du et al., 2011; Hallier et al., 1998; S. H. Kim et al., 2010; Sato et al., 2005; Uranishi et al., 2001). The relevance of this gene network in the context of ALS is not clear. In addition to interacting with transcription factors, FUS also influences gene expression through regulation of RNAP2 (RNA Polymerase II; Bertolotti, Lutz, Heard, Chambon, & Tora, 1996; Bertolotti et al., 1998; Immanuel et al., 1995; J. C. Schwartz et al., 2012) and RNAP3 (RNA Polymerase III; Tan & Manley, 2009b). FUS associates with RNAP2 (J. C. Schwartz et al., 2012) and its complex partners, such as TFIID (Bertolotti et al., 1996) and TFIIIB (Tan & Manley, 2009b), which results in either activation or repression of specific target genes. The ability of FUS to interact with and regulate the transcriptional activity of RNA polymerases suggests a general role for FUS in cellular transcriptional regulation. In fact, chromatin immunoprecipitation of HeLa lysates via antibodies directed against FUS followed by promoter microarray analyses revealed that FUS may function as a general regulator of transcription by directly binding DNA in promoter regions. The study found that FUS bound to 1,161 promoter regions (*p* < .05) for genes involved in various cellular processes, including gene expression, cell cycle, and neuronal functions (Tan et al., 2012). FUS shows specificity for binding to single-strand motifs, with the greatest affinity for a sequence in which the complementary strand forms a G-quadruplex (Figure 2(c)). Interestingly, many of the promoters bound by FUS contain sequences that are predicted to form G-quadruplexes (Tan et al., 2012). G-quadruplexes are just one of the many secondary structures formed in DNA that are evolutionarily conserved at promoters and telomeres, suggesting they may have important functions involving transcription and genome stability (reviewed in Bochman et al., 2012).

Despite the fact that FUS binds more than one thousand DNA promoters, genome-wide expression array analyses reveal only modest changes in mRNA abundance (Hoell et al., 2011; Lagier-Tourenne et al., 2012; Nakaya et al., 2013; Rogelj et al., 2012; also reviewed in Ling et al., 2013; Table 1). Intrastriatal injection of antisense oligonucleotides (ASOs) targeted to FUS caused a modest upregulation of 275 genes and downregulation of 335 genes in mice (Lagier-Tourenne et al., 2012). Only four genes exhibited greater than twofold increase in mRNA abundance upon knockdown of FUS, consistent with the idea of FUS playing a more influential role in gene downregulation. Genes downregulated by FUS are also enriched for FUS-binding sites within the mRNA transcripts, consistent with a direct regulation of these genes by FUS at the RNA level. Genes involved in synaptic activity fall within this category of genes that are directly regulated by FUS. It appears that an acute loss of FUS function in vivo is not sufficient to induce motor neuron disease, as knockdown of FUS expression for 2 weeks was reportedly insufficient to elicit a disease-related phenotype in mice (Lagier-Tourenne et al., 2012).

**Table 1.** Summary of investigations of FUS involvement in RNA processes.

		Investigations of FUS in RNA processes				
Publication	General description	mRNA Expression (method)	mRNA Splicing (method)	Binding targets (method)	RNA-binding specificity	Key categories identified by Gene Ontology Term analysis^a^
Hoell et al. (2011)	Comparison of FET family and mutant FUS RNA targets	FUS knockdown in HEK-293 cells (microarray)	N/A	HA-tagged WT, R521H, or R521G in HEK-293 cells (PAR-CLIP)	Introns; AU-rich stem loops (15-fold higher affinity than GGU repeat)	RNAs uniquely bound by mutant FUS: endoplasmic reticulum and ubiquitin-proteasome related
Colombrita et al. (2012)	Comparison of FUS and TDP-43 RNA targets	N/A	N/A	Cytoplasmic fraction of NSC-34 (RIP-CHIP)	3′UTR; limited sequence specificity	RNAs bound by FUS: transcriptional regulation, cell cycle, ribonucleoprotein biogenesis, RNA splicing, stress response/DNA repair, purine ribonucleotide binding, and ubiquitin-mediated proteolysis
Ishigaki et al. (2012)	RNA-binding specificity of FUS; expression and splicing regulation by FUS	FUS knockdown in primary cortical neurons (exon array)	FUS knockdown in primary cortical neurons (exon array)	Mouse cerebellum (HITS-CLIP)	Introns and 3′UTR; regions with secondary structure	Changes in mRNA abundance: signaling cascades and metabolic processes Alternatively spliced mRNA: vesicle transport, neuronal impulse, and neuronal projection
Rogelj et al. (2012)	Comparison of FUS and TDP-43 RNA targets; expression and splicing regulation by FUS	*FUS−/−* embryonic mouse brain (splice junction microarray)	*FUS−/−* embryonic mouse brain (splice junction microarray)	Embryonic mouse brain (iCLIP)	Long introns; no preference for stem loops; limited sequence specificity	Alternatively spliced mRNA: cell adhesion, apoptosis, neuronal development, and axonogenesis
Lagier-Tourenne et al. (2012)	Species comparison of FUS RNA targets; comparison of targets, expression, and splicing regulation between FUS and TDP-43	FUS knockdown in adult mouse brain and spinal cord (RNA-seq)	*FUS−/−* embryonic mouse brain; FUS knockdown in adult mouse brain (splicing-sensitive microarrays)	Naïve mouse brain; nondisease human brain (CLIP-seq)	Long introns and 3′UTR; GUGGU is an enriched RNA sequence motif	RNAs bound by FUS: components of the synapse and molecules residing in neuronal projections
Nakaya et al. (2013)	Species comparison of FUS RNA targets; expression and splicing regulation by FUS	FUS knockdown in embryonic stem cell (ESC)-derived mouse neurons (RNA-seq)	FUS knockdown in ESC-derived mouse neurons (RNA-seq)	Human temporal lobe cortices; ESC-derived mouse neurons (HITS-CLIP)	Introns; limited sequence specificity	RNAs bound by FUS: synapse, cell adhesion, neuronal projection, and neuronal recognition processes
van Blitterswijk et al. (2013)	Comparison of FUS overexpression, FUS knockdown, and expression of mutant FUS on mRNA expression and splicing	FUS knockdown; overexpression of WT, R521G, or R522G in HEK-293 cells (RNA-seq)	FUS knockdown; overexpression of WT, R521G, or R522G in HEK-293 cells (RNA-seq)	N/A	N/A	Changes in mRNA abundance: ribosome, spliceosome, mismatch repair, and DNA replication

*Note.* PAR-CLIP = photoactivatable ribonucleoside-enhanced cross-linking and immunoprecipitation; RIP-Chip = RNA-binding protein immunopurification microarray; HITS-CLIP = high-throughput sequencing of RNA isolated by cross-linking immunoprecipitation; iCLIP = individual-nucleotide resolution cross-linking and immunoprecipitation; CLIP-seq = cross-linking immunoprecipitation, high-throughput RNA sequencing (RNA-seq); FUS = fused in sarcoma; N/A = not applicable; HA = hemagglutinin; FET = FUS, EWS, TAF15; WT = wild type.

a Gene categories that are highlighted in the original work; see original publication for a full list of Gene Ontology terms.

## FUS in the RNA World

Dysregulation of RNA is emerging as a pathogenic mechanism in ALS and related disorders (van Blitterswijk & Landers, 2010). To determine whether RNA dysregulation is involved in disease pathogenesis, it is first necessary to elucidate the normal RNA processing functions of FUS and other disease-associated RNA-binding proteins. Recent efforts have focused on identifying RNA transcripts that interact with FUS and the factors that drive these interactions.

### RNA Motifs Recognized by FUS

FUS is also known as hnRNP (heterogenous ribonuclear protein) P2, describing its role as an RNA-binding protein (Calvio et al., 1995). Early SELEX (systematic evolution of ligands by exponential enrichment) and EMSA (electrophoretic mobility shift assays) analyses demonstrated that recombinant FUS selectively binds RNAs containing a GGUG motif with nanomolar affinity in vitro (Lerga et al., 2001). However, recent RNA cross-linking and deep-sequencing studies aimed at identifying mRNAs bound by FUS in vivo have generated mixed results (Table 1). While one report confirms an enrichment of GUGGU-rich sequences bound by FUS in naïve mouse and nondiseased human brains (Lagier-Tourenne et al., 2012), others report limited sequence specificity (Rogelj et al., 2012) and propose that FUS is selective for a conformational motif (Colombrita et al., 2012; Hoell et al., 2011; Ishigaki et al., 2012; Nakaya et al., 2013) as suggested by previous studies (Fujii & Takumi, 2005; Takahama et al., 2013). One study demonstrated that FUS binds AU-rich stem loops, an RNA structural motif, with 15-fold higher affinity than a GGU repeat RNA (Hoell et al., 2011). Therefore, FUS is able to bind GU-rich sequences in vitro and in vivo, but it appears that such sequences are neither sufficient nor required for interactions between FUS and RNA.

A consistent finding across most RNA cross-linking and deep-sequencing studies is the binding of FUS to long introns (Hoell et al., 2011; Ishigaki et al., 2012; Lagier-Tourenne et al., 2012; Rogelj et al., 2012). That FUS binds to introns within prespliced RNA is consistent with the nuclear localization of this protein and the role of FUS in splicing (discussed below). FUS exhibits preferential binding toward the 5′ end of long introns, indicative of FUS deposition on nascent transcripts during transcription elongation (Lagier-Tourenne et al., 2012; Rogelj et al., 2012). FUS-binding sites were also identified within the 3′UTR of target genes (Lagier-Tourenne et al., 2012). These sites were enriched for cytoplasmic FUS variants (Hoell et al., 2011) and for the cytoplasmic fraction of endogenous FUS (Colombrita et al., 2012).

### RNA-Binding Domains Within FUS

The FUS protein contains a single RRM, domain that is generally known for binding RNA but that also mediates interactions with DNA and proteins (Clery et al., 2008). It is often assumed that mutagenesis of four phenylalanine residues (F305L, F341L, F359L, and F368L) within the RRM of human FUS effectively abolishes interactions between FUS and RNA. Support for this notion was provided in a study wherein ultraviolet cross-linking of FUS to endogenous RNA was reduced upon mutation of these four phenylalanine residues in *Drosophila* and murine neuronal (N2A) cells (Daigle et al., 2013). Interestingly, the FUS RRM is structurally similar to other RRMs, adapting a canonical β1 -α1-β2-β3-α2-β4 fold. However, the amino acid sequence is divergent from other RRMs. Unique features of the FUS primary structure include two evolutionarily conserved lysine residues within an extended loop connecting α1 and β2, termed the *KK-loop* (Liu et al., 2013). Nuclear magnetic resonance (NMR) titration and surface plasmon resonance (SPR)-binding studies demonstrated that the binding between FUS- and GGUG-containing RNA sequences as well as single-stranded and double-stranded DNA depends on charge–charge interactions through this KK-loop. This mode of nucleic acid binding by FUS is distinct from other RRMs, which utilize classical ring stacking between aromatic (e.g., phenylalanine) residues and bases of nucleic acids (Liu et al., 2013).

Although the RRM is an obvious domain to investigate RNA-binding interactions of FUS, there are additional motifs within FUS that also bind RNA. Experiments with isolated FUS domains expressed recombinantly (Iko et al., 2004) or in mammalian cell culture (Bentmann et al., 2012) do not support a strong interaction between RNA and the FUS RRM, but instead point to the RGG1/2 and Zn finger domains for mediating tight-binding interactions between FUS and RNA. An NMR titration study reported by Iko et al. (2004) failed to detect the binding between FUS RRM and a GGUG-containing RNA sequence that was reported by Liu et al. (2013). However, this discrepancy could be explained by the low concentrations of RNA and FUS employed by Iko et al., which were below the dissociation constant for this relatively weak binding interaction (146–260 μM; Liu et al., 2013). Rather, Iko et al. demonstrated that the Zn finger region of FUS bound GGUG-containing RNA with a dissociation constant of ∼10 μM. This is in agreement with a recent report demonstrating that the region in FUS encompassing the RGG1, RGG2, and Zn finger domains (termed the *Z-domain*) bound to UG12 RNA, whereas no interaction was detected between UG12 and FUS RRM (Bentmann et al., 2012). Collectively, these observations suggest that FUS–RNA interactions are more complex than previously thought. In fact, it may be the case that multiple domains of FUS contribute simultaneously to recognize mRNA (Fujii & Takumi, 2005; J. C. Schwartz et al., 2013). One could speculate that the mode of RNA binding by FUS is context dependent with respect to the RNA and cellular condition. Regardless of the exact roles of these FUS domains in binding and processing RNA, it should not be assumed that point mutations in the RRM nor deletion of the RGG domains by themselves are sufficient to abolish all interactions between FUS and RNA.

### RNA Targets of FUS

Prior to the recent wave of RNA cross-linking and deep-sequencing studies aimed at identifying RNA transcripts bound by FUS, Fujii and Takumi (2005) reported the binding of FUS to mRNAs encoding actin-related proteins such as β-actin and Nd1-L within mouse brain extracts (Fujii & Takumi, 2005). FUS bound the 3′UTR of mRNA encoding Nd1-L, an actin-stabilizing protein. The authors proposed that FUS recognizes multiple sites or conformations within this 3′UTR, but that this interaction lacks sequence specificity (Fujii & Takumi, 2005). The microtubule-associated protein Tau (*MAPT*) is another cytoskeletal protein that has been consistently associated with FUS (Ishigaki et al., 2012; Lagier-Tourenne et al., 2012; Orozco et al., 2012; Rogelj et al., 2012). The potential roles of FUS in modulating actin dynamics and functions of the cytoskeleton are intriguing in light of ALS-linked mutations within other actin-binding proteins such as profilin-1 (Wu et al., 2012) and the impairment of axonal transport by ALS-linked proteins such as SOD1 (Cu,Zn-superoxide dismutase 1; Morfini et al., 2009).

Recent genome-wide approaches have aimed to identify all transcripts bound and potentially regulated by FUS (reviewed in Ling et al., 2013; Table 1). One PAR-CLIP (photoactivatable ribonucleoside-enhanced cross-linking and immunoprecipitation) analysis compared transcripts bound by WT FUS and two ALS-linked FUS variants (R521G and R521H) that were predominately expressed in the cytoplasm of HEK-293 cells. Thousands of transcripts were cross-linked to WT FUS as well as to FUS variants. Interestingly, 80% of transcripts bound by mutant FUS were also bound by WT FUS. The authors propose that transcripts bound exclusively by FUS variants result from the cytoplasmic mislocalization of FUS variants and not because the ALS-linked mutations themselves physically alter the binding between FUS and RNA, supporting a gain of toxic function for mutant FUS with respect to RNA binding and processing (Hoell et al., 2011). Gene categories related to proteostasis, including the unfolded protein response (UPR) and endoplasmic reticulum (ER), as well as protein binding and mitochondrion were overrepresented amongst transcripts uniquely bound by cytoplasmic FUS variants in this study (Hoell et al., 2011). However, UPR-associated transcripts were also reportedly bound to WT FUS in an RNA immunoprecipitation and chip (RIP–CHIP) analysis in mouse NSC-34 cells, likely because this protocol enriched for FUS in the cytoplasmic fraction (Colombrita et al., 2012). Additional functional categories and pathways for FUS mRNA targets in NSC-34 cells included regulation of transcription, cell cycle, ribosome genesis, spliceosome assembly, RNA processing, and DNA repair (Colombrita et al., 2012). Despite the different methodologies and cell types employed, Colombrita et al. (2012) reported a 63% overlap in the FUS mRNA targets between these two studies (Hoell et al., 2011). The effect of FUS on the expression of genes important for neuronal function, including synaptic genes, was revealed through similar analyses in mouse and human brain tissue and may bear more relevance to neurodegenerative disorders caused by FUS (Lagier-Tourenne et al., 2012). Additional mRNA targets of FUS that may be relevant to ALS and FTLD include those encoding SOD1, medium and heavy chains of neurofilament (NEFL, NEFM, NEFH), glutamate transporter (EAAT2), ubiquilin 1 and 2, and the FUS protein itself (Lagier-Tourenne et al., 2012). Importantly, a comparison of FUS mRNA targets in mouse versus human brain revealed a relatively high degree (69%) of overlap, indicating that the FUS–RNA interactomes are conserved between these species (Lagier-Tourenne et al., 2012).

The binding of FUS to its own transcript suggests an autoregulatory mechanism for FUS expression (Lagier-Tourenne et al., 2012; Nakaya et al., 2013) that may be relevant to ALS pathogenesis. Y. Zhou, Liu, Liu, Ozturk, & Hicks (2013) recently demonstrated that FUS regulates splicing of exon 7, but that this splicing activity is impaired for FUS variants that mislocalize to the cytoplasm. A misregulation of FUS expression may in turn contribute to the pathogenic accumulation of FUS in disease. This is only one recent example of FUS functioning as a splicing factor, as a role for FUS in splicing was suggested from earlier observations that FUS associates with components of the spliceosome (Kameoka et al., 2004; Meissner et al., 2003; Yang et al., 1998) and regulated 5′-splice site selection in E1A pre-mRNA (Hallier et al., 1998; Lerga et al., 2001). The global effect of FUS on alternative splicing has been revealed recently through several genome-wide exon array analyses (reviewed in Ling et al., 2013; Table 1). An Affymetrix Mouse Exon array on primary cortical neurons with knocked-down FUS expression identified more than 3,202 exons that were altered, many associated with genes having neuronal functions or linked to neurodegeneration (Ishigaki et al., 2012). Increased exon inclusion for genes involved in neuronal development was also detected in E18 *FUS−/−* mouse brains compared with WT FUS brains (Rogelj et al., 2012). Significant changes in the splicing patterns of ribosome- and spliceosome-related genes were also reported in nonneuronal cells (van Blitterswijk et al., 2013), demonstrating that FUS likely plays a general role in splicing in various cell types. Several exon array analyses in neurons and neuronal tissues (Ishigaki et al., 2012; Lagier-Tourenne et al., 2012; Rogelj et al., 2012) confirmed exon inclusion of *MAPT*, which encodes the protein tau, when FUS expression is knocked down (Orozco et al., 2012). FUS is believed to promote skipping of exons 3 and 10 in *MAPT*, whereas inclusion of exon 10 leads to FTLD and Parkinsonism (Orozco et al., 2012). Therefore, the splicing of *MAPT* by FUS may serve to protect against neurodegeneration.

With thousands of mRNAs either bound or processed by FUS, how do we determine which interactions, if any, are most relevant to ALS pathogenesis? Now that a substantial amount of big data has been collected by these genome-wide analyses, the next task is to validate hits, determine whether these genes are dysregulated in disease, and assess whether disease-related phenotypes can be rescued by restoring their regulation. This may be particularly challenging, given that the combined reduction of several targets by a loss of FUS function may contribute to disease (Lagier-Tourenne et al., 2012).

### Transport and Local Translation of RNA by FUS

One functional outcome of FUS binding to RNA is the transport of RNA from the nucleus to the cytoplasm and throughout the cell. Although FUS is predominantly expressed in the nucleus of most cells, it shuttles between the nucleus and cytoplasm (Zinszner et al., 1997). Using heterocells, Zinszner et al*.* (1997) demonstrated that nucleocytoplasmic shuttling of FUS is functionally linked to the transport of mRNA from the nucleus to cytoplasm. FUS is localized to the nucleus through a C-terminal nuclear localization signal (NLS; Figure 1), which binds the nuclear import receptor Transportin (or karyopherinβ2; Dormann et al., 2010). This interaction is modulated by methylation of arginine residues within and proximal to the NLS (Dormann et al., 2012). Arginine-methylation, catalyzed by protein arginine methyltransferases (PRMT), is a posttranslational modification that regulates the subcellular localization and function of proteins (Bedford & Clarke, 2009). Several reports have recently emerged demonstrating the effect of arginine methylation on the cellular localization of endogenous and ALS-linked FUS proteins, with a consistent finding that nuclear export of FUS requires arginine methylation (Dormann et al., 2012; Du et al., 2011; Sama et al., 2013; Tradewell et al., 2012; Yamaguchi & Kitajo, 2012).

FUS associates with several motor proteins, including the ATP-dependent actin-binding motors Myo5A (Yoshimura et al., 2006) and Myo6 (Takarada et al., 2009), and it has also been isolated as part of the large granule that associates with the microtubule-dependent kinesin motor protein KIF5B (Kanai et al., 2004). The involvement of FUS with such transport machinery and the transport of FUS to different regions of the cell may be important for local translation (Fujii et al., 2005; Fujii & Takumi, 2005; Yasuda et al., 2013). In response to synaptic activation via the glutamate receptor mGluR5, FUS translocates into dendritic spines where it may facilitate local translation of actin-associated proteins (e.g., Nd1-L; Fujii & Takumi, 2005). A loss of FUS expression results in abnormal spine morphology and attenuated spine density in hippocampal pyramidal neurons (Fujii et al., 2005). Recently, adenomatous polyposis coli (APC)-containing RNA granules, which are located at cell protrusions and function in cell migration, were shown to contain FUS. Interestingly, these granules were translationally active, and translation of kank-2 (KN motif and ankyrin repeat domains 2) within these granules was dependent on FUS expression (Yasuda et al., 2013). The association of FUS with RNA granules that modulate translation under conditions of induced stress has emerged as an active area of research, as discussed later.

## The Ying and Yang of FUS in Stress Response

Cells try to reestablish homeostasis in response to stress. However, cell death pathways are triggered under conditions of persistent or severe stress (Fulda et al., 2010; Kultz, 2005). In either case, the cell mounts a coordinated response that involves different stages of gene expression (e.g., transcription, mRNA processing, and translation; Biamonti & Caceres, 2009; Kultz, 2005; Spriggs et al., 2010; Weake & Workman, 2010). Thus, it is not surprising that FUS could play a normal role in cellular stress response given its role in regulating key aspects of gene expression. On the other hand, several studies have demonstrated a link between ALS-FUS and cellular stress response, suggesting that mutant FUS could impair this pathway in disease.

### The Association of ALS-FUS With Stress Granules

**Figure 4.**
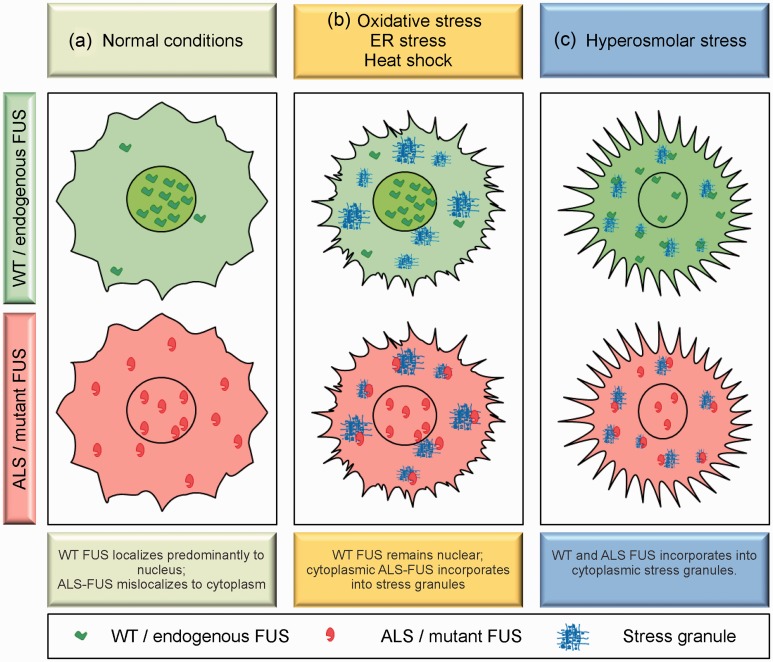
The differential response of FUS to cellular stress. Cells expressing exogenous WT or endogenous FUS (top panels) and ALS-linked mutant FUS (bottom panels) are shown under different cellular conditions. (a) Under normal conditions, WT/endogenous FUS is localized predominantly to the nucleus while ALS-FUS variants with mutations in the nuclear localization domain undergo varying degrees of cytoplasmic mislocalization. (b) Under conditions of oxidative stress, heat shock, or ER stress, WT/endogenous FUS remains nuclear while mutant FUS that is already mislocalized to the cytoplasm incorporates into stress granules. (c) Under conditions of hyperosmolar stress, WT/endogenous FUS translocates to the cytoplasm and incorporates into stress granules. Under these conditions, endogenous FUS is thought to play a prosurvival role. Mutant FUS proteins that are already mislocalized to the cytoplasm also associate with stress granules (unpublished data), although the implications of this interaction for ALS are unknown. *Note.* ER = endoplasmic reticulum; ALS = amyotrophic lateral sclerosis; WT = wild type; FUS = fused in sarcoma.

The assembly of ALS-linked mutant FUS into cytoplasmic puncta called stress granules under various conditions of applied stress has drawn considerable attention within the field over the past few years (reviewed in Bentmann et al., 2013; Y. R. Li et al., 2013; Wolozin, 2012). Stress granules are stalled translational complexes that form in response to environmental or metabolic stress (Figure 4). The proposed function of stress granules is the triage of mRNAs, dictating their fate for expression, degradation, or suppression in order to express the appropriate repertoire of proteins to reestablish homeostasis. It is becoming increasingly clear that stress granules also play a role in cellular signaling (Anderson & Kedersha, 2008; Kedersha et al., 2013). ALS-linked mutant FUS is consistently detected within stress granules under conditions of protein overexpression, oxidative stress, heat shock, and ER stress (Bentmann et al., 2012; Bosco et al., 2010; Dormann et al., 2010; Gal et al., 2011; Kino et al., 2011; Figure 4(b)). Endogenous or ectopically expressed WT FUS is generally not found in stress granules in response to these stressors, although some reports have described this observation (Andersson et al., 2008; Goodier et al., 2007; Ito et al., 2010; Kato et al., 2012; Kino et al., 2011). Rather, the association of FUS with stress granules correlates with its cytoplasmic expression, with ALS-causing variants such as FUS P525L and R495X exhibiting the most robust levels of both cytoplasmic mislocalization and stress granule incorporation. Conversely, FUS variants with nuclear expression remain nuclear and are excluded from stress granules (Bosco et al., 2010; Dormann et al., 2010). In other words, oxidative stress, heat shock, and ER stress do not induce a translocation of FUS from the nucleus to the cytoplasm (Sama et al., 2013). Rather, FUS must be in the cytoplasm and already poised to enter stress granules at the time stress is applied. In fact, WT FUS can also assemble into stress granules under these conditions when its expression is restricted to the cytoplasm (Dormann et al., 2010).

The fact that only mutant FUS robustly incorporates into stress granules under conditions of applied stress raises the possibility that mutant FUS impairs cellular stress response in ALS. Although there is no functional assay per se for stress granules, Baron et al. (2013) demonstrated that the presence of mutant FUS in stress granules altered several properties that may be important for stress granule function. Under conditions of sodium arsenite, an inducer of oxidative stress, the expression of mutant FUS delayed the assembly and expedited the disassembly of stress granules. Moreover, mutant FUS increases the dynamics of stress granules as measured by FRAP (fluorescence recovery after photobleaching). These observations are consistent with a destabilizing effect of mutant FUS on stress granules. Interestingly, the size and abundance of stress granules are enhanced by mutant FUS, which may be an outcome of the increased protein or mRNA load within these structures caused by mutant FUS (Baron et al., 2013). Intriguingly, stress granule marker proteins have been detected in pathological aggregates of postmortem tissues from individuals with ALS and FTLD (Bentmann et al., 2013; Dormann et al., 2010; H. J. Kim et al., 2013; Liu-Yesucevitz et al., 2010), suggesting that these granules may accumulate during chronic stress and thus serve as precursors to the end-stage pathological aggregates seen in these disorders (Wolozin, 2012).

Stress granules are composed of many (>50) RNA-binding proteins that contain aggregation-prone domains, including low-complexity, prion-like domains (Anderson & Kedersha, 2008; Kato et al., 2012; Y. R. Li et al., 2013). This domain in FUS (residues 1–165) facilitates aggregation in yeast (Sun et al., 2011) and drives the association of FUS with hydrogels, a biomaterial that is composed of amyloid-like fibrils and that has been proposed to mimic the physicochemical properties of stress granules (Han et al., 2012; Kato et al., 2012). Kato et al. (2012) and Han et al. (2012) demonstrated that modifications to the prion-like domain of FUS prevent the association of FUS with hydrogels and stress granules in cell culture. Conversely, other reports demonstrated a minimal contribution of the prion-like domain but rather showed the RGG domain(s) within mutant FUS directed this protein into stress granules (Baron et al., 2013; Bentmann et al., 2012). The methylation of arginine residues within the RGG domains of FUS has been proposed to modulate the assembly of FUS into stress granules. Arginine residues within the RGG domains of FUS are hypermethylated (Rappsilber, Friesen, Paushkin, Dreyfuss, & Mann, 2003), and methylated FUS is detected in postmortem aggregates of ALS patient tissues (Dormann et al., 2012). However, Baron et al. (2013) demonstrated that methylation of FUS is not a prerequisite for stress granule incorporation under sodium arsenite stress. A key question that awaits further exploration is whether FUS is recruited to stress granules through interactions with proteins, RNA, or both types of molecules.

Although the dogma in the stress granule field indicates that translation is silenced in stress granules (Kedersha & Anderson, 2002), a recent report demonstrating that FUS is present in translationally active RNA granules (Yasuda et al., 2013) raises the intriguing possibility that mutant FUS may inappropriately turn on translation in stress granules under conditions of stress. To date, however, there is no evidence that mutant FUS actually influences protein translation in either direction under conditions of stress.

### FUS, Hyperosmolar Stress Response, and ALS

While mutant FUS in stress granules supports a gain of toxic function mechanism for FUS in ALS, the discovery of a novel role for endogenous FUS in hyperosmolar stress response could provide a basis for a loss of function mechanism as well. Recently, endogenous FUS was shown to translocate from the nucleus to the cytoplasm, where it assembles into stress granules, in response to hyperosmolar stress (Sama et al., 2013; Figure 4(c)). This is in stark contrast to other stressors (e.g., overexpression, heat shock, oxidative stress, and ER stress) that do not elicit a similar response from endogenous FUS (Sama et al., 2013). The precise role of FUS in hyperosmolar stress response is not known. For example, information regarding the upstream signal(s) that trigger this response from FUS and the actions of FUS in stress granules is lacking. Importantly, reduced levels of FUS rendered cells susceptible to hyperosmolar-induced toxicity (Sama et al., 2013), indicating that FUS plays a prosurvival and protective role in hyperosmolar stress response.

Interestingly, two other ALS-linked RNA-binding proteins, TDP43 and hnRNP A1, also respond to hyperosmolar stress by translocating to the cytoplasm and incorporating into stress granules (Dewey et al., 2010; van der Houven van Oordt et al., 2000), suggesting that hyperosmolar stress may be an unacknowledged factor in ALS pathogenesis. Hyperosmolar stress triggers cell volume changes, cytoskeletal rearrangement, DNA and protein damage, cell cycle arrest, oxidative stress, and other detrimental processes that could ultimately lead to cell death (Brocker et al., 2012; Burg et al., 2007). Further, hyperosmolar stress and inflammation are tightly correlated (Brocker et al., 2012; L. Schwartz et al., 2009). As documented throughout this review, FUS has been implicated in several of the aforementioned pathways, most of which are relevant to ALS pathogenesis (Barber et al., 2011; Evans et al., 2013; Pasinelli & Brown, 2006; Rothstein, 2009; Shaw, 2006). It remains to be determined whether hyperosmolar stress contributes to ALS pathogenesis.

## Conclusions

FUS is a multifunctional protein essential for a diverse host of cellular processes, including genomic stability, RNA metabolism, and stress response. Since the discovery of FUS in ALS in 2009, there has been a substantial increase in the number of studies pertaining to the normal function of FUS, with a focus on whether ALS-causing mutations in some way alter these functions. To date, it is still unknown whether FUS causes ALS through a gain or loss of function mechanism. If it is a loss of function, which of the many functions is the culprit? Does FUS-ALS result from dysregulation of one or multiple pathways? To date, there is relatively little known about the secondary or tertiary structure of FUS in vitro or inside cells. Does mutant FUS misfold as we have seen for other ALS and neurodegenerative disease proteins? Additional insight into these questions is needed to shape our therapeutic strategies targeted to FUS-mediated ALS pathogenesis.
